# Human perivascular stem cells prevent bone graft resorption in osteoporotic contexts by inhibiting osteoclast formation

**DOI:** 10.1002/sctm.20-0152

**Published:** 2020-07-22

**Authors:** Stefano Negri, Yiyun Wang, Takashi Sono, Seungyong Lee, Ginny Ching‐Yun Hsu, Jiajia Xu, Carolyn A. Meyers, Qizhi Qin, Kristen Broderick, Kenneth W. Witwer, Bruno Peault, Aaron W. James

**Affiliations:** ^1^ Department of Pathology Johns Hopkins University Baltimore Maryland USA; ^2^ Orthopaedic and Trauma Surgery Unit, Department of Surgery, Dentistry Paediatrics and Gynaecology of the University of Verona Verona Italy; ^3^ Department of Plastic Surgery Johns Hopkins University Baltimore Maryland USA; ^4^ Departments of Molecular and Comparative Pathobiology and Neurology Johns Hopkins University Baltimore Maryland USA; ^5^ UCLA and Orthopaedic Hospital Department of Orthopaedic Surgery and the Orthopaedic Hospital Research Center Los Angeles California USA; ^6^ Center for Cardiovascular Science and MRC Center for Regenerative Medicine University of Edinburgh Edinburgh UK

**Keywords:** adipose stem cell, bone graft, mesenchymal stem cell, osteoclast, pericyte, perivascular stem cell, spine fusion

## Abstract

The vascular wall stores mesenchymal progenitor cells which are able to induce bone regeneration, via direct and paracrine mechanisms. Although much is known regarding perivascular cell regulation of osteoblasts, their regulation of osteoclasts, and by extension utility in states of high bone resorption, is not known. Here, human perivascular stem cells (PSCs) were used as a means to prevent autograft resorption in a gonadectomy‐induced osteoporotic spine fusion model. Furthermore, the paracrine regulation by PSCs of osteoclast formation was evaluated, using coculture, conditioned medium, and purified extracellular vesicles. Results showed that PSCs when mixed with autograft bone induce an increase in osteoblast:osteoclast ratio, promote bone matrix formation, and prevent bone graft resorption. The confluence of these factors resulted in high rates of fusion in an ovariectomized rat lumbar spine fusion model. Application of PSCs was superior across metrics to either the use of unpurified, culture‐defined adipose‐derived stromal cells or autograft bone alone. Under coculture conditions, PSCs negatively regulated osteoclast formation and did so via secreted, nonvesicular paracrine factors. Total RNA sequencing identified secreted factors overexpressed by PSCs which may explain their negative regulation of graft resorption. In summary, PSCs reduce osteoclast formation and prevent bone graft resorption in high turnover states such as gonadectomy‐induced osteoporosis.

## Graphical abstract

Perivascular stem cells (PSCs) derived from adipose tissue prevent bone graft resorption and enhance arthrodesis in a preclinical osteoporotic spinal fusion model. PSCs inhibit osteoclasts formation via paracrine secretion of negative regulators in a high bone turnover environment.

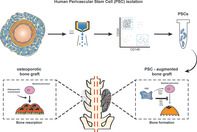

**Significance statement**

Perivascular progenitor cells exert positive regulatory effects on osteoblasts to heal bones, yet their potential role in osteoclast regulation is not known. It is observed that human perivascular progenitor cells reduce osteoclast formation, thereby preventing bone graft resorption and yielding better outcomes in a preclinical xenograft model. In the future, perivascular stem cells could be used to augment bone grafts, serving as a pro‐anabolic, antiosteoclastic stimulus for better outcomes in orthopaedics.

## 1 INTRODUCTION

As early as the 17th century, the modern technique of bone grafting has been used to repair or replace skeletal tissues. Despite the ability of bone grafts to take successfully, the primary failure of this methodology is via osteoclast mediated resorption,^1^ which can be seen in diverse clinical scenarios from spine fusion^2^ to cleft palate repair.^3^ Bone grafts are particularly prone to resorption in states of high bone turnover,^4^ such as chronic kidney disease,^5^ rheumatoid arthritis,^6^ corticosteroid‐induced osteoporosis, and postmenopausal osteoporosis.^7^ Indeed, the clinical entity of osteoporosis has a dramatic impact on numerous outcomes after spine fusion procedures, such as pseudoarthrosis, pedicular screws pull‐out, rod breakage, cage subsidence, proximal junction kyphosis, adjacent fractures, and persistent pain.^8^, ^9^, ^10^, ^11^ Methods to augment bone grafts by minimizing chances of bone graft resorption would be clinically impactful, particular in the context of high bone turnover states.

Mesenchymal progenitor cells that reside within vessel walls are multipotent,^12^, ^13^, ^14^, ^15^, ^16^, ^17^ are native progenitors of mesenchymal stem cells (MSCs), and contribute to endogenous bone repair.^18^, ^19^ When purified according to the expression of CD146 (Mel‐CAM) and CD34, human perivascular stem cells (PSCs) from adipose tissue or other tissue compartments hasten bone repair.^20^, ^21^, ^22^, ^23^, ^24^, ^25^ Despite direct differentiation of human PSCs toward chondroblasts, osteoblasts, and osteocytes,^25^ PSCs induce bone healing primarily via paracrine stimulation of resident host cells within the bone tissue.^21^, ^26^ Osteoinductive paracrine effects are either via elaboration of nonvesicular proteins, such as bone morphogenetic proteins,^22^, ^27^, ^28^ or via secretion of bioactive extracellular vesicles (EVs).^29^ In contrast, various and contradictory effects of mesenchymal progenitor cells on osteoclasts have been reported.^30^, ^31^, ^32^, ^33^ However, the role of purified PSCs in regulating osteoclastic formation or function has not been investigated.

Here, we describe an effective and clinically translatable approach to augment osteoporotic bone grafts with human perivascular progenitor cells. Briefly, PSCs when mixed with autograft bone induced an increase in osteoblast:osteoclast ratio, induced bone matrix formation, and prevented bone graft resorption. The confluence of these factors resulted in high rates of spine fusion in a gonadectomy‐induced osteoporosis model. Within in vitro experiments, we determined that PSCs negatively regulate osteoclast formation, and do so via nonvesicular paracrine factors.

## 2 MATERIALS AND METHODS

### 2.1 Human PSC purification from adipose tissue

PSCs were isolated from human subcutaneous adipose tissue via fluorescence‐activated cell sorting (FACS) as previously reported.^24^, ^29^, ^34^, ^35^ Human lipoaspirates were obtained under IRB approval at Johns Hopkins University with a waiver of informed consent (protocol number IRB00119905). Fat tissue was stored for less than 48 hours at 4°C before processing. The stromal vascular fraction (SVF) of human lipoaspirate was obtained by enzymatic digestion.^35^ The resulting SVF was further processed for cell sorting, using a mixture of the following directly conjugated antibodies: anti‐CD34‐allophycocyanin (1:100; BD Pharmingen, San Diego, California), anti‐CD45‐allophycocy‐anin‐cyanin 7 (1:100; BD Pharmingen), anti‐CD146‐fluorescein isothiocyanate (1:100; Bio‐Rad, Hercules, California), and anti‐CD31‐allophycocyanin‐cyanin 7 (1:100, Bio‐Rad). All incubations were performed at 4°C for 15 minutes. Summary of antibodies is presented in Supplementary Table S1. In this manner, a combined population of pericytes (CD146^+^CD34^−^CD45^−^CD31^−^) and adventitial cells (CD34^+^CD146^−^CD45^−^CD31^−^) was isolated to constitute the PSC population. Sorted PSCs were either applied in a rat spinal fusion model, or culture expanded for in vitro studies. For in vitro expansion, cells were cultured at 37°C in a humidified atmosphere containing 95% air and 5% CO_2_. PSCs were cultured in DMEM (ThermoFisher Scientific, Inc, Waltham, Massachusetts), 10% fetal bovine serum (FBS, ThermoFisher Scientific, Inc.), 1% penicillin/streptomycin. Medium was changed every 3 days unless otherwise stated.

### 2.2 Animals and conditions

Female 12‐week‐old athymic rats were used (strain code 316, Charles River Laboratories Inc, Wilmington, Massachusetts), which exhibit essentially normal bone repair.^36^, ^37^, ^38^ Experimental procedures were consistent with ethical principles for animal research and were approved by Johns Hopkins University IACUC (protocol number RA19M268). Throughout the study, rats were single housed in an IVC system rack using polypropylene cages (37 cm × 25 cm × 24 cm), with 12/12 night/day cycles, 21°C (±2°C) and 50% (±20%) relative humidity. All rats had ad libitum access to complete rat food and filtered water. Animal allocation is described in Supplementary Tables S2 and S3.

### 2.3 Osteoporosis induction and assessment of bone mass

Animals were ovariectomized through a dorsal bilateral approach to induce osteoporosis.^39^, ^40^ To perform ovariectomy (OVX), animals were anesthetized with inhaled isoflurane (3% induction, 2% maintenance) delivered with combined oxygen and nitrous oxide (1:2 ratio) along with subdermal injection of sustained‐release buprenorphine (1.2 mg/kg SC, q72h) and enrofloxacin (5 mg/kg). A 10‐mm longitudinal skin incision was made at the costovertebral area bilaterally. The peritoneal cavity was explored, and bilateral ovaries excised. Peritoneum and skin were then closed with 4‐0 resorbable sutures (Ethicon Inc, Somerville, New Jersey). Dual‐energy x‐ray absorptiometry (DXA) based assessment for bone mineral density (BMD) was performed to confirm bone mass loss postoperatively, performed every 4 weeks after OVX, using a UltraFocus Faxitron equipment (Faxitron Bioptics, Tucson, Arizona). BMD was measured considering a lumbar spine region of interest (ROI) encompassing the L3 to L6 vertebral bodies.^41^ In addition, body weight was recorded every 4 weeks.

### 2.4 Osteoporotic bone graft preparation and cell supplementation

**FIGURE 1:**
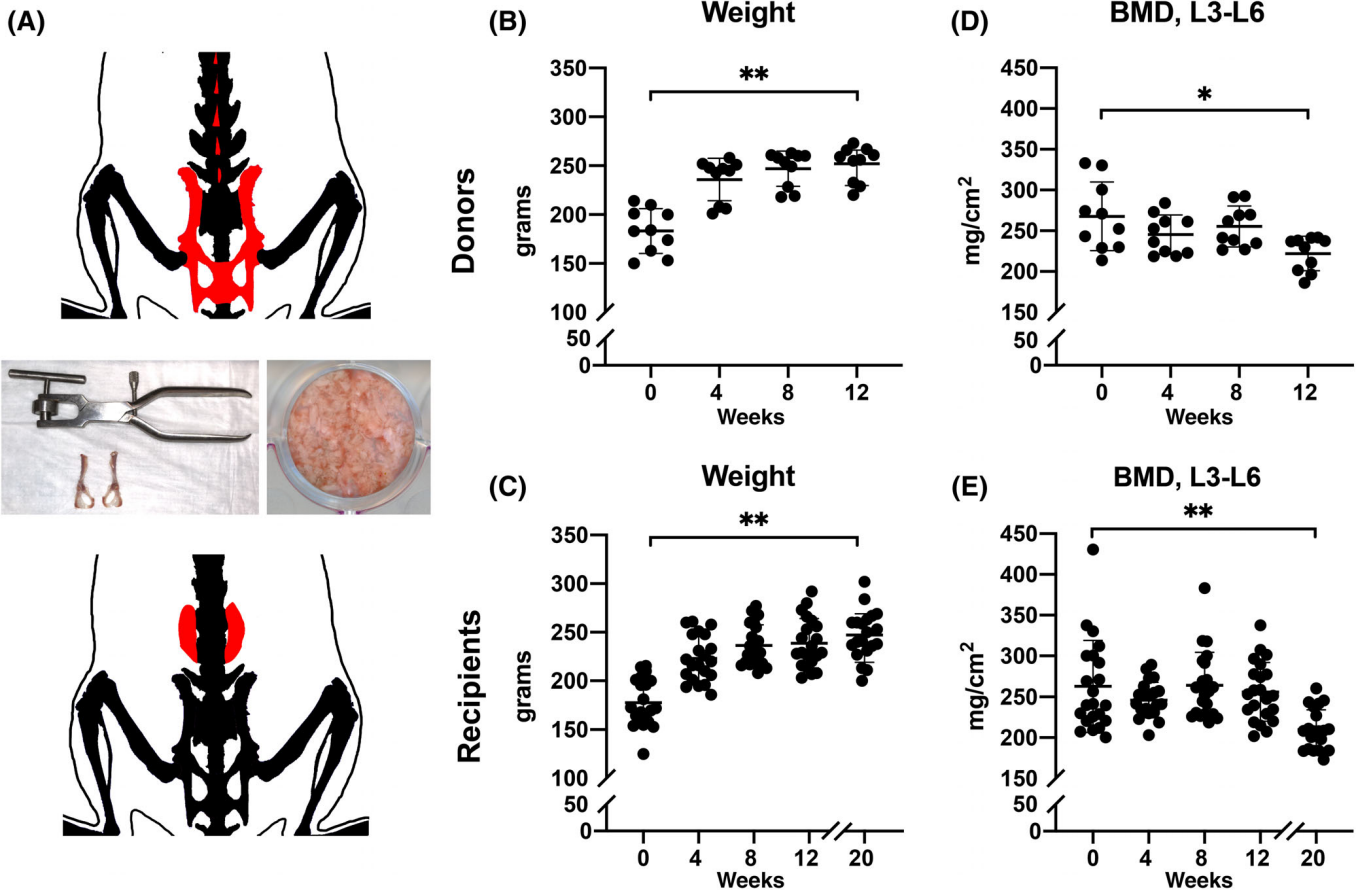
Graft preparation for posterolateral spine fusion, and validation of gonadectomy‐induced osteoporosis. A, Schematic illustration of bone graft harvesting (above), preparation of graft with bone morselizer (middle), and placement in the surgical area (below). The red areas indicate regions of corticocancellous bone for harvest and placement in the paravertebral lumbar (L) spaces between L4 and L5. B,C, Growth curves of (B) donor and (C) recipient animals for bone grafting. Zero week indicates time of ovariectomy (OVX) among donor and recipient animals. Bone grafting from donor to recipient animals was performed at 12 weeks, and spine fusion monitored at up to 20 weeks. D,E, Bone mineral density (BMD) across time, as measured by dual‐energy X‐ray absorptiometry (DXA) within a L3‐L6 region of interest among (D) donor and (E) recipient animals. N = 10 total donor rats and N = 21 total recipient rats. *P < .05 and **P < .01 in comparison to 0 week

A finely minced osteoporotic bone graft was prepared from corticocancellous pelvic and lumbar spinous apophysis bones derived from syngeneic female animals 12 weeks after OVX using a dental bone morcelizer device (G.S. online store, Seattle, Washington) (Figure 1A).^39^ A total amount of 0.60 g of bone graft was used per spinal fusion (0.30 g per side) according to previous published studies.^42^ In general, one donor provided the bone graft needed for three surgical procedures. Implants were prepared using 0.5 × 10^6^ total PSCs or adipose‐derived stromal cells (ASCs) per animal (0.25 × 10^6^ per fusion spine), adapted from our previous study.^21^ Briefly, each bone graft (0.30 g) was placed into an individual well of a 24‐well plate and evenly dispersed across the well. Next, 0.25 × 10^6^ PSCs in 500 μL DMEM were seeded onto the bone graft and incubated at 37°C for 1 to 2 hours. Cell‐bone graft interaction was characterized in vitro focusing on adhesion kinetics and cell viability.^43^ Cell adhesion to the bone graft was evaluated with scanning electronic microscopy (SEM). Briefly, samples were fixed in 2.5% glutaraldehyde, 3 mM MgCl_2_, in 0.1 M sodium cacodylate buffer, pH 7.2 overnight at 4°C. After buffer rinse, samples were postfixed in 1% osmium tetroxide in 0.1 M sodium cacodylate buffer (1 hour) on ice in the dark. Following a DH_2_O rinse, samples were dehydrated in a graded series of ethanol and left to dry overnight in a desiccator with hexamethyldisilazane (HMDS). Samples were mounted on carbon coated stubs and imaged on the Zeiss Leo FESEM (field emission scanning electron microscope) at 1 kV. Viability was evaluated with confocal imaging after Cyto‐Dye staining (MilliporeSigma, Burlington, Massachusetts) using a Zeiss 780 imaging system (Zeiss, Thornwood, New York). in vivo persistence of seeded cells was evaluated using PKH26 dye according to the manufacturer's instructions (MilliporeSigma).

### 2.5 Spinal fusion procedure

Twelve weeks after OVX, posterolateral lumbar spinal fusion was performed. Anesthesia and analgesia were the same as described above. Spinal fusion surgeries were performed as previously described.^44^, ^45^, ^46^ Briefly, a 3‐cm midline incision was carried out in order to perform a L4/L5 surgical access. The transverse processes of L4 and L5 were bilaterally exposed by blunt muscle splitting technique. High‐resolution posterioranterior radiograph was performed in order to confirm the appropriate level. Then, decortication of the L4 and L5 transverse processes with a low speed burr (Roboz Surgical Instrument Co, Gaithersburg, Maryland) was carried out under regular irrigation with sterile normal saline solution. A 0.3 g bone graft with or without PSCs was allocated bilaterally and shaped with a smooth periosteal elevator (Roboz Surgical Instrument Co) covering the dorsal aspect of the decorticated transverse processes and the area between them. Rats were sacrificed 8 weeks postoperatively via CO_2_ overdose, and the spines were harvested for analysis.

### 2.6 Experimental groups

Rats were divided into three different groups: (a) OVX‐induced osteoporotic autograft alone, (b) OVX‐induced osteoporotic autograft +0.5 × 10^6^ PSCs, and (c) an additional group with of OVX‐induced osteoporotic autograft +0.5 × 10^6^ ASCs was used as unpurified cellular control. Animal allocation is further summarized in Supplementary Table S3.

### 2.7 Assessment of spinal fusion by manual palpation

At 8 weeks postimplantation, the lumbar spine specimens were taken out en bloc. Manual palpation was performed to evaluate the reduction of motion between the lumbar spines of rats, as previously described.^47^ The samples were tested by three blinded observers and scored on a scale of 1 to 5 by flexing or extending the specimens. The scoring criteria were as follows: 1 indicates motion between vertebrae, with no bone mass formation; 2 indicates motion with a unilateral bony mass; 3 indicates motion with bilateral bony masses; 4 indicates no motion between vertebrae, with moderate bilateral bone masses bridging transverse processes; and 5 indicates no motion, with abundant bilateral bone.

### 2.8 DXA and microcomputed tomography (μCT) assessments of bone graft sites

Cell augmented bone grafts were assessed using a combination of DXA and μCT. First, the BMD of bone graft sites was prospectively analyzed every 4 weeks with DXA using regions of interest between the transverse processes of the L4 and L5 vertebrae. Second, general morphological description and morphometric analysis were performed using ex vivo microCT using a Skyscan 1275 scanner (Bruker‐MicroCT, Kontich, Belgium) with the following settings: 55 kV, 181 μA, 1 mm aluminum filter in 180°, six frames per 0.3° with a 20‐μm voxel size. Images were reconstructed using NRecon. DataViewer software was used to realign the images and quantitative parameters were assessed using Skyscan CTan software (SkyScan, Kontich, Belgium) as previously published.^21^, ^45^ Briefly, polygonal ROIs were outlined including the minced bone graft and the newly formed bone matrix between the L4 and L5 transverse processes. Preexisting bone structures were excluded. A threshold value range of 61 to 255 was used. After global thresholding was carried out, a three‐dimensional (3D) data analysis including bone volume, bone volume/tissue volume, bone surface, trabecular thickness, trabecular spacing, and trabecular number was performed.

### 2.9 Histology and immunohistochemistry

Specimens were fixed in 4% paraformaldehyde for 48 hours, decalcified with 14% ethylenediaminetetraacetic acid for up to 2 months and embedded in the optimum cutting temperature compound. Sections were prepared at 10‐μm thickness with a Cryofilm type 3c microtome (SECTION‐LAB, Hiroshima, Japan). Sections were stained with hematoxylin and eosin (H&E) and Goldner's trichrome. Tartrate‐resistant acid phosphatase (TRAP) and alkaline phosphatase (ALP) were used as functional enzymatic methods using previously published methods.^48^ Histomorphometric parameters related to osteoclasts and osteoblasts were measured using the OsteoMeasure system (OsteoMetrics, Atlanta, Georgia)^49^ and following the nomenclatures described by the American Society for Bone and Mineral Research Nomenclature Committee guidelines.^50^ For immunofluorescence staining, sections were washed with phosphate buffered saline (PBS) three times, 10 minutes each. Antigen were retrieved with a trypsin enzymatic antigen retrieval solution (ab970; Abcam, Cambridge, Massachusetts). All sections were blocked with 5% goat serum in PBS for 1 hour at 25°C. Primary anti‐osteocalcin (OCN) antibodies were added to each section (1:100) and incubated overnight at 4°C. Next, Alexa Fluor 647‐conjugated secondary antibodies (1:200) were used. Sections were counterstained and mounted in DAPI mounting medium (H‐1500, Vector laboratories, Burlingame, California). Images were obtained on a Leica DM 6B microscope (Leica Biosystems, Lincolnshire, Illinois).

### 2.10 Osteoclastogenesis assays

In order to assay the paracrine effects of PSCs on osteoclast formation, bone marrow monocyte/macrophage lineage cells (BMMs) were first harvested from 8 to 10 weeks old wild‐type male mice by flushing the marrow space of femora and tibiae. The flushed bone marrow cells were cultured in α‐minimum essential medium (α‐MEM) containing 10% FBS, 1% penicillin/streptomycin, and 20 ng/mL macrophage colony‐stimulating factor (M‐CSF) (R&D Systems, Minneapolis, Minnesota) for 3 days. BMMs were then incubated in 48‐well plates (5 × 10^4^ cells per well) with 10 ng/mL M‐CSF and 50 ng/mL RANKL (R&D) for 5 days. Next, TRAP staining was performed on cultured osteoclasts using a commercially available kit (Sigma‐Aldrich) prior fixation with PFA 4% for 10 minutes. After osteoclast differentiation, TRAP‐positive multinucleated cells containing more than three nuclei were identified as osteoclasts. ImageJ was used to quantify total osteoclasts number and area per field of view (×4).^51^ All experiments were performed in triplicate, with analyses performed in a blinded fashion.

PSC biological effect on osteoclastogenesis was assessed with three different methods, including (a) coculture, (b) application of PSC conditioned medium (CM), or (c) application of PSC‐derived EVs. For coculture assays, BMMs were seeded at 1 × 10^5^ cells/well in a 24‐well plate, and PSCs were seeded at 10000 or 20 000 cells per Transwell insert, using inserts with a 0.4 μm pore size (Corning Inc, Kennebunk, Maine). For CM assays, PSCs were seeded in a 10 cm dish and cultured with growth medium. After confluence, cells were washed three times with PBS and medium was replaced by DMEM without FBS and cultured for additional 24 hours. The resulting CM was collected and either applied during osteoclast differentiation (at a concentration of 2% or 5% CM), or further processed for EV purification, as previously published.^29^ Briefly, EVs were isolated by serial centrifugation at 300*g* for 10 minutes, 2000*g* for 30 minutes, 10 000*g* for 30 minutes, and 120 000*g* for 4 hours at 4°C. The resulted EV pellet was resuspended in PBS. EV isolates were validated as previously published^29^ and in accordance with guidelines set forth by the International Society for Extracellular Vesicles (ISEV)^52^ using a combination of size distribution evaluation using nanoparticle tracking analysis (Nanosight), visualization of EVs with transmission electron microscopy, and western blot to confirm enrichment in tetraspanin molecules but without cellular contaminants (CD9, CD63, CD81, calnexin).^29^ PSC‐EV were applied to BMMs during osteoclast differentiation at concentrations based on our prior reports in other cell types.^29^

### 2.11 Transcriptomics

The RNA content of PSC‐EVs and parent PSCs was detected by total RNA sequencing as previously described.^29^ Briefly, total RNA was extracted from PSCs by Trizol (Life Technologies Corporation, Carlsbad, California). PSC‐EV‐derived RNA was purified using exoRNeasy Serum Plasma Kits (Qiagen, Hilden, Germany) following the manufacturer's guidelines. The RNA samples were quantified by deep sequencing with the Illumina NextSeq 500 platform (Illumina, San Diego, California). Data were analyzed using software packages including CLC Genomics Server and Workbench (RRID:SCR_017396 and RRID:SCR_011853), Partek Genomics Suite (RRID:SCR_011860), Spotfire DecisopnSite with Functional Genomics (RRID:SCR_008858).

### 2.12 Statistical analysis

Statistical analysis was performed using an appropriate analysis of variance to analyze more than two groups, followed by a post hoc Tukey's test. A Fisher's exact test was used to analyse categorical variables such as fusion score analysis. The statistical software, GraphPad Prism 8.1 Version (GraphPad Software, San Diego, California) was used for all statistical analyses. *P < .05 and **P < .01 were considered significant.

## 3 RESULTS

### 3.1 Validation of low bone mass after OVX

In order to best mimic bone autografting in osteoporotic conditions, syngeneic athymic female rats were used to prepare the donor bone graft (Figure 1). Corticocancellous bone was harvested from rats 12 weeks after OVX, and bone graft was standardly prepared and applied in a posterolateral spinal fusion procedure to syngeneic animals that were also 12 weeks post‐OVX (Figure 1A). Body weight and lumbar BMD were monitored every 4 weeks after OVX (Figure 1B‐E). Both donor and recipient ovariectomized animals had a similar starting weight and growth curve (Figure 1B,C, mean starting weight: 183.2 ± 23.0 and 177.8 ± 24.4 g for donor and recipient rats, respectively). Likewise, mean lumbar BMD was similar between donor and recipient rats at the study outset (Figure 1D,E, 267.7 ± 42.2 and 262.6 ± 56.3 mg/cm^2^, respectively). As expected, both donor and recipient rats demonstrated a significant decline in lumbar BMD after OVX (mean reduction in lumbar BMD of 45.7 ± 40.0 and 52.5 ± 47.7 mg/cm^2^ among donor and recipient animals, respectively). These findings confirmed the overall similarity of donor and recipient athymic rats, and the achievement of a low bone mass state in all groups after gonadectomy.

### 3.2 Validation of bone graft supplementation with human PSCs

**FIGURE 2:**
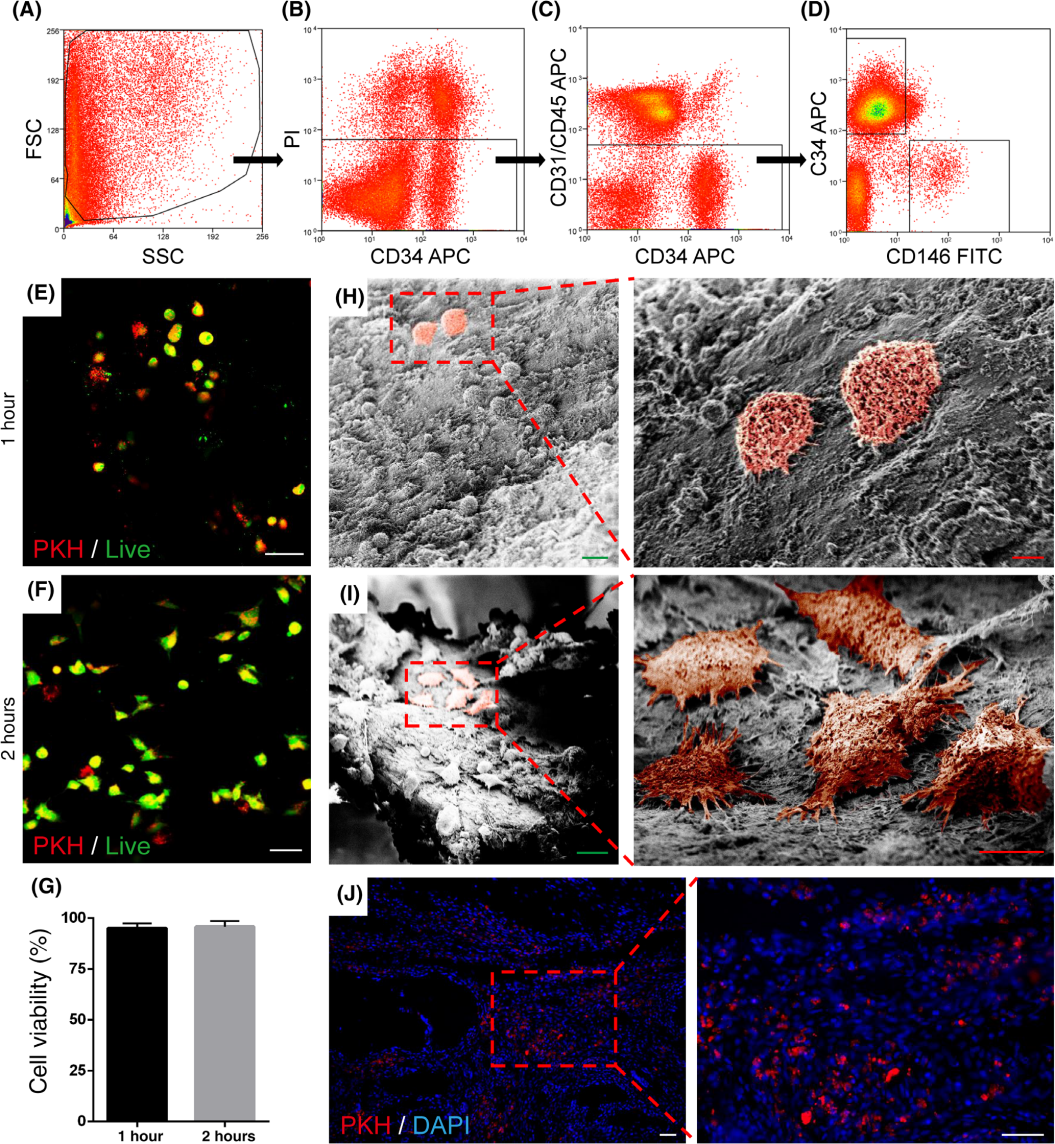
Human perivascular stem cell (PSC) derivation and validation of cell seeding on autograft bone. A‐D, Purification of human PSCs via FACS, including (A) size characterization, (B) removal of PI positive nonviable cells, (C) removal of endothelial and hematopoietic cells, and (D) isolation of perivascular stem cells, including CD146^+^CD34^−^ pericytes and CD146^−^CD34^+^ adventitial cells. E,F, Viability of PKH pre‐labeled PSC (appearing red) when seeded on bone graft at 1 and 2 hours, as assessed by live cell staining (appearing green). Overlap appears yellow. G, Quantification of (E,F) calculated as % cell viability among PKH‐labeled PSC. H,I, Kinetics of PSC adhesion to bone graft, evaluated with scanning electronic microscopy (SEM) at 1 and 2 hours. PSC are colorized red. J, in vivo persistence of PKH labeled‐PSCs, 2 weeks after spinal fusion. All experiments performed in triplicate. Graph represent mean and error bars represent one SD. White scale bar = 50 μm; green scale bar = 20 μm; red scale bar = 10 μm

Our approach was to improve osteoporotic bone graft outcomes by the supplementation of human PSCs. As our prior studies have used polymers^25^ or demineralized bone matrices^21^, ^24^, ^53^ for cell delivery, we first attempted to optimize the seeding and viability of PSCs when placed on rat corticocancellous bone grafts. PSCs were isolated from fresh human lipoaspirates as previoulsy described,^22^, ^29^, ^35^ as a bipartite population of FACS purified pericytes (CD34^−^CD146^+^CD31^−^CD45^−^), and adventitial cells (CD34^+^CD146^−^CD31^−^CD45^−^) (Figure 2A‐D). For the present study, PSCs represented 34% of total SVF (1% pericytes, 34% adventitial cells). PSC viability and attachment onto prepared athymic rat bone graft was next assessed (Figure 2E‐J). Prior to seeding, PSCs were labelled with the PKH26 fluorescent dye. To further confirm viability, Cyto‐dye staining was performed at 1 and 2 hours after seeding on the graft material (Figure 2E,F, appearing green). Nearly all PKH‐labelled PSCs were also stained with Cyto‐dye (dual positive cells appearing yellow). Quantification of photographic images demonstrated 95.1% and 95.9% viability of seeded PSCs at 1 and 2 hours, respectively (Figure 2G).

These initial fluorescence imaging studies also suggested that the morphology of PSCs changes from 1 to 2 hours post seeding from a more rounded to a spindled and stellate appearance. To further investigate, SEM was performed at 1 and 2 hours after PSC seeding (Figure 2H,I). At 1 hour, seeded cells demonstrated a more rounded shape with few cytoplasmic extensions toward the bone surface (Figure 2H, PSCs appear red). At 2 hours, seeded PSCs showed clear features of attachment to bone surfaces, with seeded cells flattened and spread across bone surfaces with numerous attachment points (Figure 2I). These data suggested that a 2 hours incubation period would be of benefit for PSC‐bone graft attachment, but without impairing cell viability.

We next validated that PSCs survive when added to bone graft in the early postoperative period after spinal fusion. PKH‐labeled PSCs were seeded on osteoporotic bone grafts, and implanted in our lumbar spine fusion model. After 2 weeks, the bone graft site showed numerous PKH^+^ PSCs (Figure 2J). Having validated PSC viability, attachment, and survival on corticocancellous bone grafts, we next sought to examine their efficacy in preventing graft resorption.

### 3.3 Human PSCs prevent bone graft resorption and improve posterolateral spine fusion

In our experimental study, three groups were compared, including (a) bone graft alone (control), (b) bone graft supplemented with human PSCs, and (c) as a further comparison, bone graft supplemented with equivalent numbers of unpurified, culture‐defined ASCs. See Supplementary Table S2 for a summary of animals used, and Supplementary Table S3 for a further description of treatment groups, cell numbers, and bone graft composition.

**FIGURE 3:**
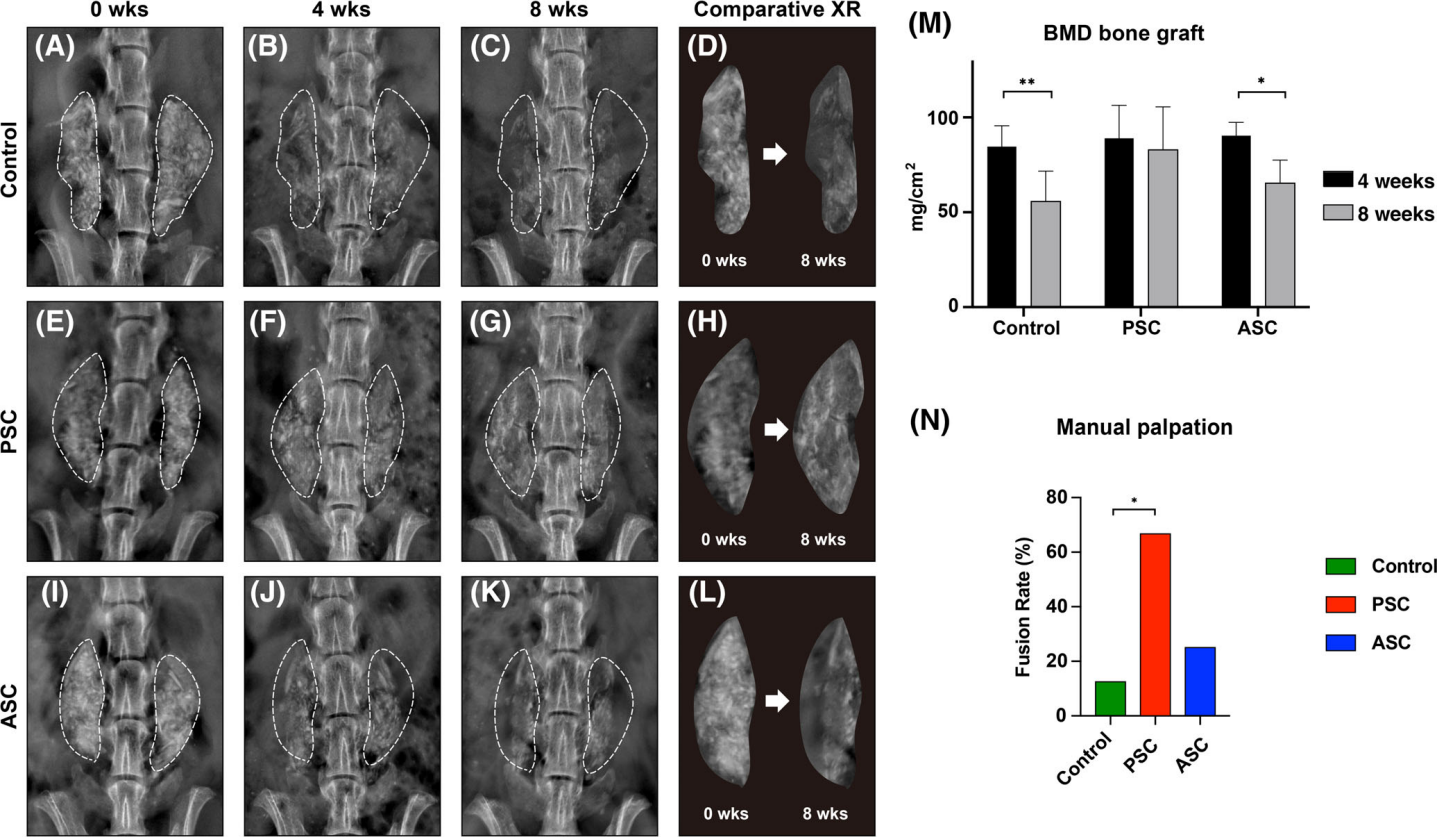
Human perivascular stem cells (PSCs) prevent bone graft resorption and promote spine fusion in athymic rats. A‐L, High‐resolution roentgenography (XR), immediately postoperatively (0 week, far left), at 4 weeks postoperative (middle left), 8 weeks postoperative (middle right), as well as the illustrative change from over the course of 8 weeks (far right column). Dashed white lines indicate bone graft site. A‐D, Representative appearance of acellular control (bone graft alone). E‐H, Representative appearance of human PSC augmented bone grafts. I‐L, Representative appearance of human ASC augmented bone grafts. M, Bone mineral density (BMD) of the bone graft site, as determined by dual‐energy X‐ray absorptiometry (DXA) at 4 and 8 weeks postoperative. Graph represent mean and error bars represent 1 SD. N, Mean spine fusion rate at 8 weeks postoperative, as determined by manual palpation scoring. See Supplementary Tables S2 and S3 for a summary of animal numbers. See Supplementary Table S3 for a summary bone graft weight and total cell numbers. *P < .05; **P < .01

High‐resolution roentgenography was performed every 4 weeks, and showed that bone graft controls without cell augmentation exhibited overall an increasing radiolucency from 0 to 8 weeks postoperatively (Figure 3A‐D). In contrast, bone grafts supplemented with PSCs tended to have a preserved radioopacity across time (Figure 3E‐H). Finally, bone grafts supplemented with ASCs showed a pattern more similar to the bone graft controls, again with an increasing radiolucency from 0 to 8 weeks (Figure 3I‐L). This qualitative change was further analysed using DXA specifically of the bone graft site at 4 and 8 weeks postoperatively (Figure 3M). Here, bone grafts without cell supplementation showed a 43% reduction in BMD (**P < .01). Likewise, ASC‐augmented bone grafts showed a 31% reduction in BMD (*P < .05). In contrast, PSC‐augmented bone grafts showed no significant reduction in BMD.

At the study endpoint, spine fusion was assessed using a previously validated grading system, by manually applying flexion/extension forces to assess intervertebral motion at the L4‐L5 level (Figure 3N). Results showed that PSC‐augmented bone grafts yield a fusion rate of 66.7%. This is compared to a fusion rate of 12.5% among rats treated with bone grafts alone, and 25% among rats treated with ASC‐augmented bone grafts. In summary, PSC‐treated bone grafts showed minimal resorption, which was associated with a significantly higher incidence of spine fusion.

### 3.4 Human PSCs improve μCT metrics of bone grafts

**FIGURE 4:**
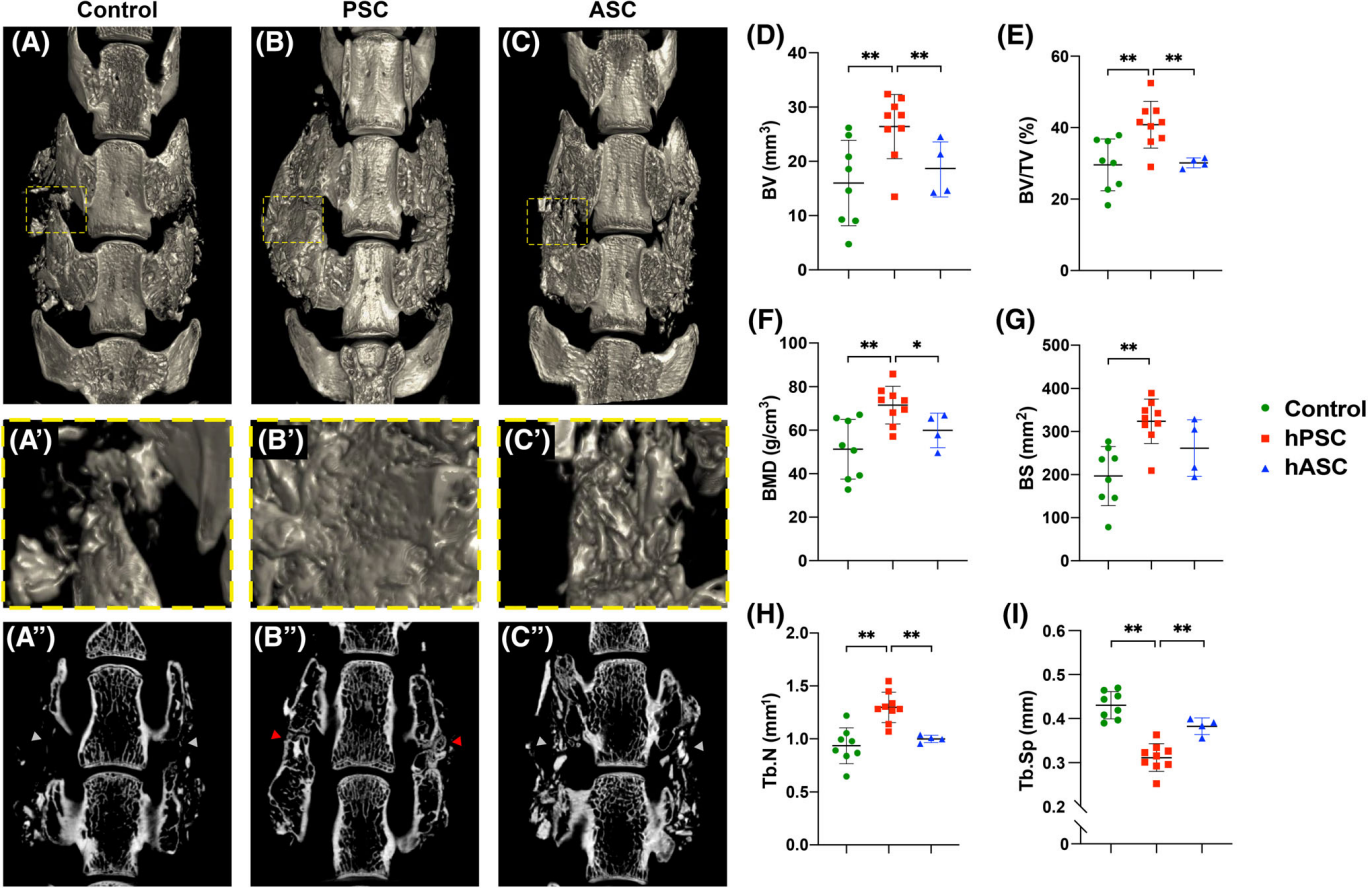
Human perivascular stem cells (PSCs) induce robust bone formation in osteoporotic spine fusion as assessed by microcomputed tomography (μCT). A‐C, Representative three dimensional (3D) μCT reconstructions of spine fusion site at 8 weeks post‐operative, shown from a dorsal perspective. A′‐C′, High‐magnification inset to show area of bridging between adjacent lumbar levels. A″‐C″, Coronal sections at level of the transverse processes to evaluate spinal fusion. Bone bridging between transverse processes is shown by red arrowheads. Lack of bone bridging in control groups is shown by grey arrowheads. D‐L, Quantitative μCT analysis of bone graft sites, including (D) bone volume (BV), (E) fractional bone volume (BV/TV), (F) bone mineral density (BMD), (G) bone surfaces (BS), (H) trabecular number (Tb.N), (I) trabecular spacing (Tb.Sp), and (J) trabecular thickness (Tb.Th). Dots in scatterplots represent an individual animal, while crosshairs and whiskers represent mean and 1 SD, respectively. *P < .05; **P < .01

Cell augmented bone grafts were next assessed using high‐resolution μCT imaging at the study endpoint (Figure 4). Three‐dimensional μCT reconstructions of the lumbar spine showed larger bony masses in PSC‐augmented spine fusion sites in comparison to either acellular control or ASC‐augmented bone grafts (Figure 4A‐C). Coronal cross‐sectional μCT images confirmed frequent bony bridging among PSC‐augmented spine fusion sites in comparison to other groups (compare red and grey arrowheads, Figure 4A″‐C″). These radiographic findings were confirmed using quantitative μCT analysis across all animals, including analysis of bone volume (BV), bone volume density (BV/TV), BMD and bone surfaces (Figure 4D‐G). Here, PSC‐augmented bone grafts resulted in a 34.0% to 56.7% increase in quantitative metrics in comparison to acellular controls (*P < .05; **P < .01). Similarly, PSC‐augmented bone grafts showed significantly increased BV, BV/TV, and BMD in relation to ASC‐augmented bone grafts (18.0%‐36.0% increase across measured parameters). In agreement with other μCT findings, trabecular bone analysis showed similar results, including an increase in trabecular number (Tb.N) and reduction in trabecular spacing (Tb.Sp) among PSC‐augmented bone graft sites (Figure 4H,I).

### 3.5 Human PSCs uncouple osteoblast and osteoclast formation

**FIGURE 5:**
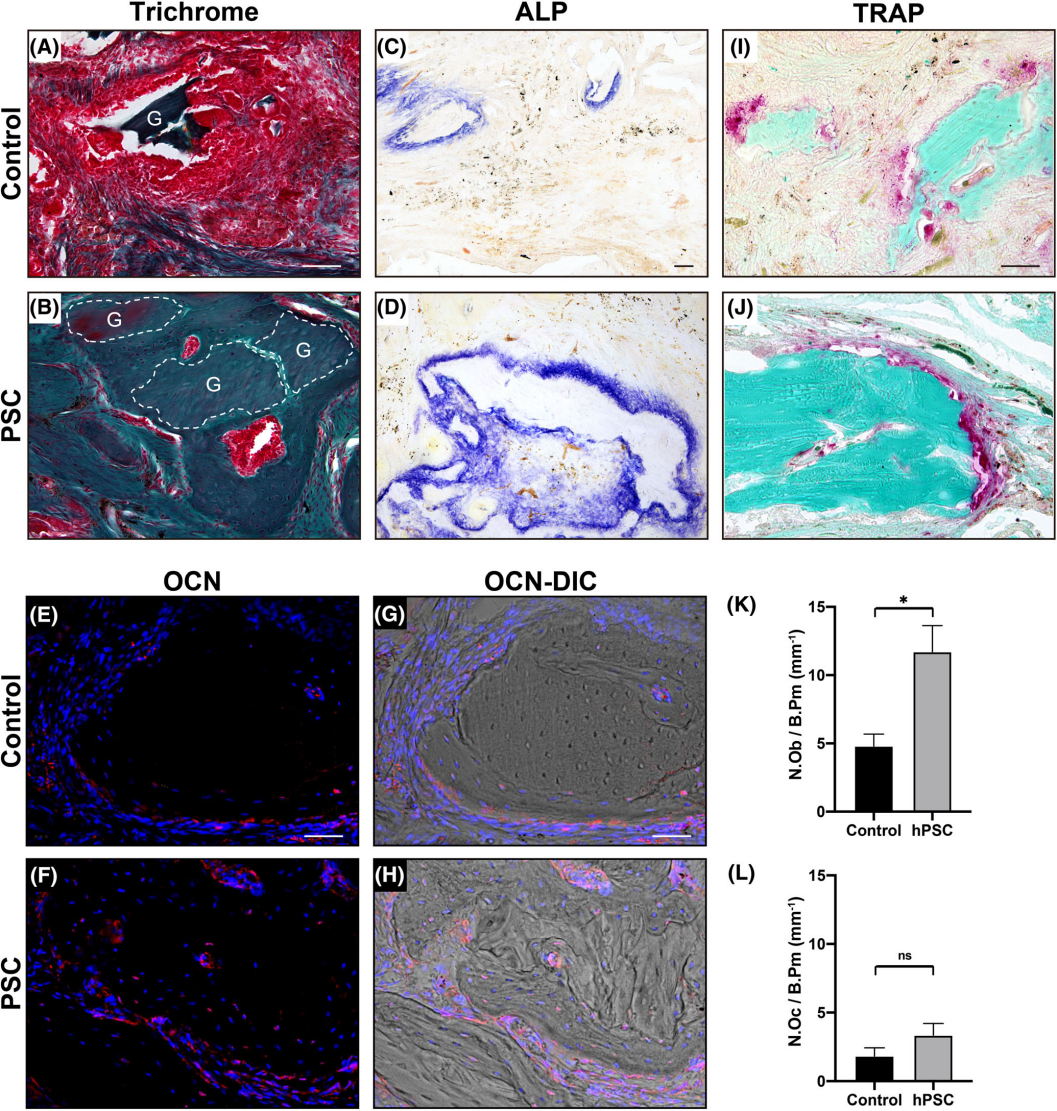
Human perivascular stem cells (PSCs) induce osteoblast formation without increased osteoclast activity during osteoporotic spine fusion. All analyses performed at 8 weeks postimplantation. A,B, Modified Goldner's trichrome staining among control‐treated or PSC‐treated spine fusion segments. Bone matrix appears blue/green, while fibrous tissue appears red. “G” denotes bone graft material. C,D, Alkaline phosphatase (ALP) staining among control‐treated or PSC‐treated spine fusion segments, appearing blue. I,J, Tartrate resistant acid phosphatase (TRAP) staining among control‐treated or PSC‐treated spine fusion segments, appearing red. Bone appears green with fast green counterstain. E,F, Osteocalcin (OCN) immunohistochemical staining, appearing red. DAPI nuclear counterstain appears blue. G,H, Corresponding OCN immunostaining, shown in DIC. K, Quantification of osteoblast numbers per bone perimeter (N.Ob/B.Pm) within control‐ or PSC‐treated bone graft sites. L, Quantification of osteoclast numbers per bone perimeter (N.Oc/B.Pm) within control‐ or PSC‐treated bone graft sites. White and black scale bars = 50 μm. *P < .05; ns, not significant

Histological analyses were next performed on spine fusion sites, which further confirmed morphologic differences associated with PSC‐augmented bone grafting. Goldner's trichrome staining within the control group showed residual bone graft material in a hypocellular fibrous background, with minimal newyl formed woven bone (Figure 5A,B). In contrast, in PSC‐treated groups, incorporation of the bone graft material was more evident, including newly formed woven bone and continuity between bone graft and the native transverse processes. Histochemical staining for ALP showed more intense areas of osteoblastic activity among bone‐lining cells within PSC‐augmented bone graft sites (Figure 5C,D). Likewise, increased detection of the osteoblast specific marker OCN was found among PSC‐augmented bone grafts (Figure 5E‐H). Osteoclast activity was assessed on TRAP stained sections (Figure 5I,J). Histomorphometric analysis of bone graft sites confirmed our observations regarding the uncoupled nature of osteoblast and osteoclast numbers within PSC‐augmented bone graft sites (Figure 5K,L). A 143.8% increase in osteoblast numbers per bone perimeter (N.Ob/B.Pm) was found among PSC‐augmented bone graft sites (Figure 5K). In contrast, no significant difference in the number of osteoclasts per bone perimeter (N.Oc/B.Pm) was detected between groups (Figure 5L).

### 3.6 Human PSCs inhibit osteoclast formation by nonvesicular paracrine means

**FIGURE 6:**
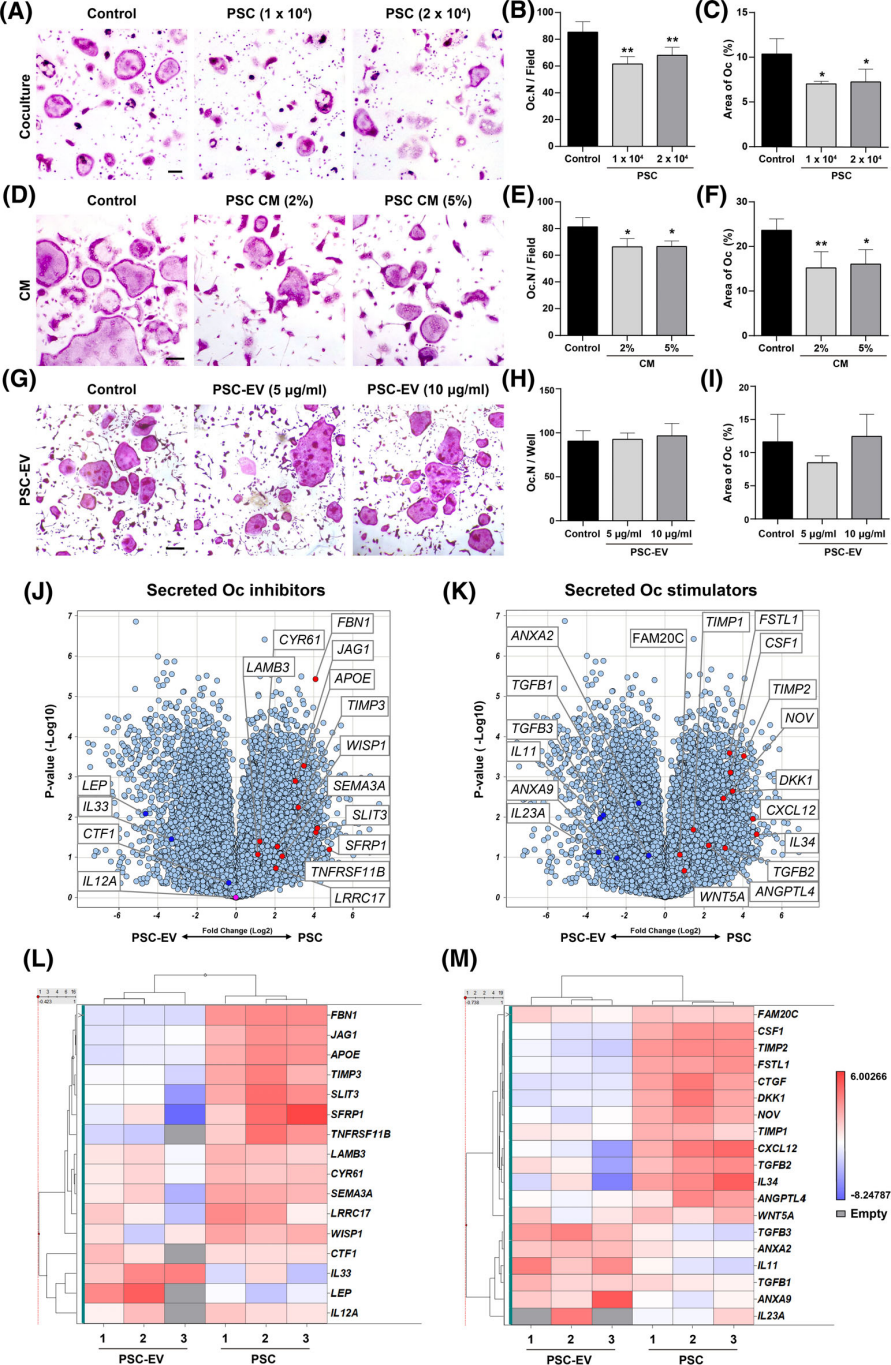
Perivascular stem cells (PSCs) inhibit osteoclastogenesis via paracrine means. The paracrine effects of PSCs on bone marrow monocyte/macrophage lineage cells osteoclastogenesis was examined using three separate techniques. All experiments were performed using induction medium including M‐CSF (10 ng/mL) and RANKL (50 ng/mL) for 5 days. A‐C, Osteoclast formation assays under noncontact coculture conditions with PSCs. Assessed with either 1 × 10^4^ or 2 × 10^4^ PSC per well. A, Representative TRAP staining after 5 days. B, Quantification of osteoclast numbers (Oc.N) and (C) Oc Area. D‐F, Osteoclast formation assays using PSC conditioned medium (CM), including (D) representative TRAP staining, (E) Oc.N, and (F) Oc Area. G‐I, Osteoclast formation assays with PSC‐derived extracellular vesicles (PSC‐EV, 5‐10 μg/mL), including (G) representative TRAP staining, (H) Oc.N, and (I) Oc Area. J‐M, Total RNA Sequencing comparison of PSC and their matched PSC‐EV isolates. N = 3 PSC and PSC‐EV samples analyzed. J,K, Volcano plots showing differential gene expression among PSC‐EV and their respective parent cells (PSC). Red dots indicate transcripts enriched in PSC, whereas blue dots indicate transcripts enriched in PSC‐EV. J, Genes encoding secreted, osteoclast (Oc) inhibitory proteins. K, Genes encoding secreted, osteoclast (Oc) stimulatory proteins. L, Heat map demonstrating differential mRNA expression levels of osteoclast inhibitory factors among three PSC‐EV preparations in relation to their parent cells. M, Heat map demonstrating mRNA expression levels of osteoclast stimulatory factors among three PSC‐EV preparations in relation to their parent cells. All experiments performed in experimental triplicate. Scale bar = 100 μm. *P < .05; **P < .01 in comparison to control

The paracrine effects of PSCs on osteoblast formation have been previously documented by our group.^30^ However, a regulatory effect on osteoclasts and bone resorption has not been previously investigated. To further investigate, three sets of in vitro osteoclast differentiation studies were performed. First, PSCs were placed in noncontact coculture conditions with BMMs in osteoclast differentiation conditions (Figure 6A‐C). PSC coculture led to a significant reduction in osteoclast numbers (Oc.N) and Oc area (27.9%‐35.7% reduction). Next, parallel experiments were performed using PSC CM (Figure 6D‐F). Osteoclast formation in the presence of PSC CM was likewise reduced (18.5%‐32.2% reduction in the presence of 2% or 5% PSC CM).

Recently, we observed that EVs derived from PSCs retain many of the biologic features of their parent cells, including pro‐osteoblastogenic effects.^29^

PSC‐derived EVs (PSC‐EVs) were next examined for potential direct inhibition of osteoclast formation. As in our prior report, PSC‐EVs were derived by ultracentrifugation, and their yield and purity confirmed^29^ prior to application, and in accordance with ISEV criteria.^52^ In contrast to coculture or CM assays, PSC‐EV treatment led to no observable effect on osteoclastogenesis (Figure 6G‐I).

Results thus far suggested that OC inhibitory factors are present within parent PSCs but not PSC‐derived EVs. To begin to investigate this phenomenon, a previously derived total RNA sequencing dataset was further interrogated (Figure 6J‐M).^29^ Here, three separate human PSC isolates were examined in comparison to their respective purified EVs. Transcripts were normalized by fragments per kilobasepair per million mapped (FPKM), and those with Log2 FPKM > −0.8 were further analyzed. Among these, 10 256 annotated genes were expressed in all samples of 54 136 total RNA transcripts (19% of total).^29^ Next, potential osteoclast regulatory factors were assessed within this dataset, including genes encoding secreted osteoclast inhibitory or activating factors (Figure 6J‐M). Negative regulators of osteoclast differentiation were differentially expressed among PSCs and PSC‐EVs, as shown by volcano plots (Figure 6J) and heatmaps (Figure 6L). A handful of such genes were enriched within PSCs, including the decoy receptor for RANKL osteoprotegerin (*TNRSF11B*), the Wnt and RANKL inhibitor secreted frizzled‐related protein‐1 (*SFRP1*),^54^ and antiosteoclastic/axonal guidance molecules such as semaphorin 3A (*SEMA3A*)^55^ and slit guidance ligand 3 (*SLIT3*).^56^ Other OC regulatory genes such as *IL12A* ^57^ were expressed to a similar degree by both PSCs and PSC‐EVs. Positive regulatory factors of OC differentiation were also differentially expressed between PSCs and PSC‐EVs (Figure 6K,M). For example, colony stimulating factor 1 (*CSF1*) and follistatin 1 (*FSTL1*)^58^ were enriched among PSCs, while other osteoclastogenic factors such as *IL11* were overrepresented among PSC‐EVs.^59^ In summary, PSC exert inhibitory effects of osteoclastogenesis via noncontact dependent effects, which appear to be via EV‐independent effectors.

## 4 DISCUSSION

In summary, our observations suggest that human perivascular progenitor cells once isolated from their native vasculature have negative regulatory effects on osteoclast formation via EV‐independent paracrine mechanisms. These observations have therapeutic relevance, and may be used to prevent bone graft resorption, especially in high bone turnover contexts. These new observations add to the established paracrine effects that perivascular progenitor cells exert within skeletal tissue, including mitogenic, pro‐migratory, and osteoblastogenic effects on osteoprogenitors,^29^ as well as immunomodulatory effects.^60^

EVs and exosomes are of increasing therapeutic interest in relation to mesenchymal stem/progenitor cells and regenerative medicine. We recently observed that PSC‐derived EVs, including exosomes and microvesicles, directly stimulate skeletal progenitor cells.^29^ Also observed in this paper, PSC‐EVs showed some modest differences in terms of recipient cell type,^29^ although hematopoietic cells or osteoclasts were not assessessed. Here, we observed that while PSCs negatively regulate OC formation, this is not a cellular effect shared with their EVs. Other studies have shown that bone marrow mesenchymal stem cell‐derived EVs have stimulatory effects on OCs via the induction of important osteoclast specific proteins such as *Nfatc1*, *Trap*, and *Ctsk*.^61^ Here, we observed that only the nonvesicular secretome houses antiosteoclastic properties. It is intriguing to speculate under what circumstances mesenchymal stem cell CM, which contains both EVs and non‐EV components, may be of more therapeutic usefulness as opposed to purified EVs alone.

Although the exact nonvesicular OC inhibitory factors are still unknown, our study identified a number of candidates. Noteably, primary modulators of osteoclastogenesis such as *TNF Receptor Superfamily Member 11b* (*Osteoprotegerin*) as well as *Colony Stimulating Factor 1* (*CSF1*) are both significantly more expressed in PSC compare to PSC‐EVs but they cannot be considered as the only underlying factors. Our RNA sequencing results indentified other potential PSC‐enriched factors. An example is the extracellular matrix protein fibrillin 1, which acts to sequester RANKL and antagonize NFATc1 signalling.^62^ Another interesting finding is enrichment for *ApoE* expression among PSCs. This gene encodes a protein with a key role in lipid metabolism that can also inhibit osteoclasts through downregulation of c‐Fos, NFATc1, and nuclear factor‐kappa B signaling.^63^ Counterbalancing this, PSC also demonstrated expression of *CXCL12*, a member of CXC chemokine family, which is a known stimulatory cofactor for osteoclast development and function.^64^

There are several limitations to our results. First, our observations regarding a negative regulatory effect on bone graft resorption were found in a postmenopausal (postgonadectomy) osteoporosis model. It will be interesting to determine if different scenarios of low bone mass, such as senile osteoporosis, or steroid‐induced osteoporosis would yield a similar outcome with PSC‐augmented bone grafting. Conversely, it is not clear if the same beneficial effect would occur in young animals. This is an important consideration, as bone grafting procedures are common in the pediatric population. Second, we specifically analyzed how PSCs induce paracrine effects on osteoclast formation in vitro. However, the identity of the exact nonvesicular secreted factors that negatively regulate OCs, whether one or multiple, has not been precisely defined. We anticipate that further study examining PSC CM using a combination of proteomics and neutralizing antibodies would represent a logical next step in this line of inquiry. Finally, it is not clear if PSCs only influence early OC formation, or if PSCs have negative regulatory effects on OC activity as well.

## 5 CONCLUSION

In summary, PSCs reduce osteoclast formation via nonvesicular paracrine mechanisms and prevent bone graft resorption in high turnover states such as gonadectomy‐induced osteoporosis. These data solidify the pleiotropic paracrine effects of PSCs on skeletal cells, and suggest the utility of such cells for cell‐augmented bone grafting to prevent surgical failure.

## CONFLICT OF INTEREST

Bruno Peault is the inventor of perivascular stem cell related patents held by the UC Regents. Aaron W. James is a paid consultant for Novadip, and receives funding for unrelated research from MTF Biologics and Novadip. This arrangement has been reviewed and approved by the Johns Hopkins University in accordance with its conflict of interest policies. Kristen Broderick declared advisory role with Margin Probe. The other authors delared no potential conflicts of interest.

## AUTHOR CONTRIBUTIONS

S.N., Y.W.: collection and/or assembly of data, data analysis and interpretation, and manuscript writing; T.S., S.L., C.‐Y.H., J.X.: collection and/or assembly of data, data analysis and interpretation; A.W.J.: manuscript writing, final approval of manuscript, conception and design, financial support; K.B., K.W.W., B.P.: provision of study materials.

## Supporting information


**Supplementary Table S1** Antibodies used.
**Supplementary Table S2.** Allocation of athymic rats
**Supplementary Table S3.** Description of the experimental groups

## Data Availability

The data that support the findings of this study are available on request from the corresponding author.
