# Supplementary material for: Human perivascular stem cells prevent bone graft resorption in osteoporotic contexts by inhibiting osteoclast formation
Source: Stem Cells Transl Med. 2020 Jul 22;9(12):1617–30. doi: 10.1002/sctm.20-0152 (PMC7695633; doi:10.1002/sctm.20-0152)
Supplement: Supplementary file 1 — Supplementary Table S1 Antibodies used. Supplementary Table S2. Allocation of athymic rats Supplementary Table S3. Description of the experimental groups [file SCT3-9-1617-s001.docx]

**Supplementary Informations**

**Supplementary Tables**

**Supplementary Table S1.** Antibodies used.

| **Antibody** | **Company** | **Catalog #** | **Use** |
| --- | --- | --- | --- |
| Mouse anti-Human CD31 | BD Pharmingen | 563653 | FACS |
| Mouse anti-Human CD45 | BD Pharmingen | 557833 | FACS |
| Mouse anti-Human CD34 | BD Pharmingen | 555824 | FACS |
| Mouse anti-Human CD146 | Bio-Rad | MCA2141 | FACS |
| Anti-Osteocalcin (OCN) | Abcam | ab93876 | IF |
| Goat anti-Rabbit AF647 | Abcam | ab150079 | IF |

FACS: Fluorescent activated cell sorting; IF: Immunofluorescent staining.

**Supplementary Table S2.** Allocation of athymic rats

| **Study phase** | **Donor #** | **Recipient #** | **Total** |
| --- | --- | --- | --- |
| Validation (*in vitro* & *in vivo*) | 2 | 1 | 3 |
| Experimental (*in vivo*) | 8 | 20 | 28 |
| **Total animals** | | | **31** |

**Supplementary Table S3.** Description of the experimental groups

| **Group** | **Scaffold** | **Total Cells # / animal** | **Total bone graft / animal** | **Timepoint** | **Animal #** |
| --- | --- | --- | --- | --- | --- |
| Control | Osteoporotic bone graft | - | 0.60 g | 8 weeks | 8 |
| hPSC | Osteoporotic bone graft | 0.5 x 10^6^ | 0.60 g | 8 weeks | 9 |
| hASC | Osteoporotic bone graft | 0.5 x 10^6^ | 0.60 g | 8 weeks | 4 |
